# The Emerging Role of Sialic Acids in Obesity and Diabetes: Molecular Mechanisms and Therapeutic Perspectives

**DOI:** 10.3390/biom15111522

**Published:** 2025-10-29

**Authors:** Xinyi Peng, Haojun Li, Qingwen Wang, Peng George Wang, Yang Ji

**Affiliations:** 1 Department of Pharmacology, School of Medicine, Southern University of Science and Technology, Shenzhen 518055, China; xinyipeng1221@gmail.com (X.P.); 12333000@mail.sustech.edu.cn (H.L.); wangp6@sustech.edu.cn (P.G.W.); 2 Department of Rheumatism and Immunology, Peking University Shenzhen Hospital, Shenzhen 518036, China; 3 Key University Laboratory of Metabolism and Health of Guangdong, Southern University of Science and Technology, Shenzhen 518055, China; 4 The Key Laboratory of Inflammatory and Immunology Diseases, Shenzhen 518036, China

**Keywords:** sialic acids, sialyltransferase, obesity, diabetes, neuraminidase, Siglec, insulin resistance, IgG glycosylation, GM3 ganglioside

## Abstract

Sialic acids are terminal monosaccharides that cap glycans on glycoconjugates. Accumulating clinical and experimental evidence shows that obesity, insulin resistance, and diabetes are accompanied by changes in sialic-acid levels. In these conditions, the sialic-acid axis is also broadly remodeled: writers (sialyltransferases), erasers (neuraminidases), and readers (Siglecs) are dysregulated across adipose tissue, liver, pancreas, endothelium, and blood, shifting insulin signaling and inflammatory tone. This review summarizes relevant studies from the perspectives of disease clinical indicators, molecular mechanisms, and interventions targeting sialic acid. Taken together, these results confirm that sialic acids and related molecules play important roles in multiple metabolic diseases; however, controversies remain due to differences in glycan structure, isoforms, and tissue specificity, particularly regarding the precise roles of neuraminidases. Future studies should build on advanced, standardized glycomic and glycoproteomic measures to define molecule- and tissue-specific roles of sialic acids in metabolic disease, enabling reliable biomarkers and guiding targeted therapy.

## 1. Introduction: The Emerging Role of Sialic Acids in Metabolic Disease

**Figure 1:**
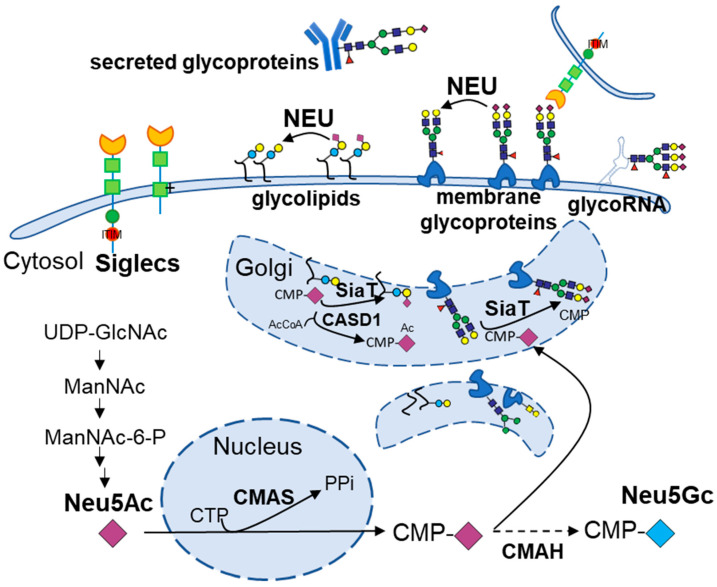
Biosynthesis, turnover, and Siglec-interactions of sialylated glycoconjugates. De novo Neu5Ac is produced from UDP-GlcNAc in the cytosol, activated in the nucleus by CMP-sialic acid synthetase (CMAS) to CMP-Neu5Ac, and transported into the Golgi, where sialyltransferases (SiaT) cap the glycans on nascent glycoproteins or glycolipids. The cytidine monophosphate-N-acetylneuraminic acid hydroxylase (CMAH) converts CMP-Neu5Ac to CMP-Neu5Gc. The enzyme CASD1 mediates the 9-O-acetylation of sialic acids. Cell-surface sialoglycans engage inhibitory or activating Siglecs to modulate signaling. Neuraminidases (NEUs) can remove terminal sialic acids either on cell surface glycoproteins/glycolipids or in the lysosome.

Sialic acids, a structurally heterogeneous family of nine-carbon terminal monosaccharides, are mandatory modulators of numerous biological processes, including cellular communication, immune response, and pathogen recognition [1]. These monosaccharides are typically situated at the outermost positions of glycoconjugates, including glycoproteins and glycolipids, either on the mammalian cell surface or extracellularly secreted (Figure 1). They are a significant component of the glycocalyx, a dense shield glycan layer that mediates cell-to-cell and cell-to-environment contacts [2]. RNAs carrying terminal sialoglycans (glycoRNAs) serve as a novel and less abundant component of the glycoconjugate [3]. While the function of glycoRNA remains under investigation, emerging evidence suggests potential roles in immune recognition [4,5]. The most abundant and biologically significant member of the sialic acid family is N-acetylneuraminic acid (Neu5Ac or NANA) [6]. However, in most mammals (including primates except for New World monkeys), another subtype of sialic acid, N-glycolylneuraminic acid (Neu5Gc), is also expressed, whereas humans have lost the ability to synthesize Neu5Gc due to an inactivating mutation in the CMAH gene [6]. This evolutionary divergence potentially alters interactions of human sialoglycans with endogenous receptors and exogenous sialic acid–binding lectins from microbes, thereby shaping immune recognition and maintaining homeostatic balance [7,8]. The biosynthesis of sialylated glycoconjugates starts with the de novo formation of N-acetylneuraminic acid (Neu5Ac) from UDP-N-acetylglucosamine. Neu5Ac is then activated to CMP-Neu5Ac and transferred to growing glycoconjugates by sialyltransferases in the Golgi apparatus [1]. The biological roles of sialylation are further shaped by the specific glycosidic linkages, most commonly α2-3, α2-6, or α2-8, formed by different sialyltransferases, each conferring distinct recognition and signaling properties [1].

In addition, sialic acids can undergo various chemical modifications that further expand their structural heterogeneity [1]. Among these, O-acetylation is one of the most common modifications. The enzyme CASD1 mediates 9-O-acetylation, a modification that regulates receptor binding and resistance to sialidase cleavage [9]. Sialidases (neuraminidases) counterbalance sialyltransferases by removing terminal sialic acids from glycoconjugates, thereby dynamically regulating the composition and signaling properties of the sialylated glycocalyx (Figure 1). Sialic acids are recognized by a family of endogenous receptors known as Siglecs (sialic acid-binding immunoglobulin-type lectins), which are predominantly expressed on immune cells [10]. In addition to mediating adhesion and phagocytosis, most Siglecs contain immunoreceptor tyrosine-based inhibitory motifs (ITIMs) that recruit phosphatases to restrain cellular activation, thus functioning as immune checkpoints [10]. Beyond its immune regulation roles, sialylation also serves several key physiological functions. These include maintaining protein stability, mediating cellular interactions, and modulating stress responses [11]. For example, sialylation has been reported to alleviate endoplasmic reticulum (ER) stress by promoting the clearance of misfolded proteins [12]. It may also protect cells under hypoxia by supporting HIF-1α signaling, and stabilize glycoproteins under oxidative stress to prevent misfolding [13]. These baseline functions of sialylation form a necessary foundation for understanding how its dysregulation contributes to disease.

Recent advances in glycobiology, particularly in relation to metabolic disorders such as obesity and type 2 diabetes, have been significantly informed by new findings concerning the role of sialic acid. Increasing evidence suggests that dysregulation of sialylation correlates with and can contribute to the pathogenesis of metabolic syndromes. This review endeavored to cover information on the function of sialic acids, their regulatory enzymes, and Siglec receptors in obesity and diabetes. It will discuss the context-dependent relationship between sialic acids and metabolic disease, where different isoforms of sialyltransferases and sialidases exert tissue-specific effects that can either promote or protect against metabolic dysfunction. In the broader picture of glycoscience, this review will explore their potential as biomarkers and therapeutic targets.

## 2. Clinical Associations of Sialic Acids in Obesity

Multiple studies have consistently reported that serum total sialic acid (TSA) levels are usually elevated in obese individuals and reflect oxidative stress [14,15], cardiovascular risk [16,17], insulin resistance [18], and chronic systemic inflammation levels [19]. For instance, serum sialic acid increased with higher body mass index (BMI) in both men and women. It is associated with higher total cholesterol, triglycerides, and low-density lipoprotein(LDL) [14,20]. In a cohort of 257 overweight/obese women, both TSA and CRP increased with greater metabolic-syndrome burden [21]; however, only TSA remained associated after BMI adjustment. Specifically, for every 0.34 mmol/L higher TSA, the likelihood of meeting ≥3 metabolic-syndrome criteria was nearly doubled after controlling for BMI (*p* < 0.0001), whereas the CRP association was attenuated by BMI adjustment. These data indicate that TSA captures metabolic-syndrome features independently of body weight, consistent with TSA acting as a more stable integrative marker of chronic inflammation and metabolic risk than CRP [22]. In pediatric patients, serum TSA levels correlate strongly with body fat percentage in obese children [17]. Neu5Ac has also been reported in another study among a cohort of 424 Chinese children aged 6–9 years as a fecal metabolite marker positively correlated with BMI, and body fat distribution [23]. Chronic inflammation is a hallmark of obesity-related metabolic dysfunction. The rise in serum sialic acids in obesity may result from inflammation-induced shedding or secretion of sialic acids from cells or cell membranes and increased secretion of sialic acid–rich, inflammation-sensitive proteins [24].

Elevated circulating sialic acid levels have also been implicated in obesity and its complications in mouse models [25]. Suzzi et al. documented sialic acid as a key metabolite that was implicated in immune exhaustion and accelerated cognitive decline in diet-induced obese mice with Alzheimer’s disease [25]. They had found diets rich in fat to elevate Neu5Ac, which induced immune exhaustion by suppressing CD4+ T cell proliferation and increasing regulatory T cell accrual in obese mice. The rise in systemic Neu5Ac levels was associated with cognitive impairment, indicating that sialic acid might contribute to the exacerbation of obesity-related complications through immunosuppressive mechanisms [25].

In a randomized trial comparing two different approaches to calorie restriction: intermittent energy restriction and continuous energy restriction, total serum sialic acid levels had no significant differences among groups or time points [26]. Despite other improvements in metabolic health and inflammation markers, this lack of change in sialic acid levels suggests that sialic acid may not be as responsive to energy restriction interventions as other inflammatory markers like CRP. It may reflect the more stable or longer-term nature of sialic acid as an indicator of chronic inflammation, relative to more highly changing markers like CRP [26]. The research underlines the complexity of inflammatory reactions to weight loss and metabolic therapy, calling for further studies to elucidate the possible involvement of sialic acid in relation to dietary therapies and metabolic health.

Interestingly, unlike the increase in total serum sialic acid, enhanced sialylation of multiple glycoproteins has been shown to exert beneficial effects on metabolism [27,28]. Glycosylation of serum proteins is likely affected by dietary patterns. A diet aligned with the Dietary Guidelines for Americans enhances sialylation of several metabolism-related serum proteins, potentially reducing cardiometabolic risks [28]. High plasma apoC-III levels in humans are associated with hypertriglyceridemia and a high prevalence of pro-atherogenic small dense LDL particles [27]. The apoC-III proteoform containing two sialic acid residues (apoC-III2) has different effects on lipid metabolism compared with asialylated (apoC-III0) or the most abundant monosialylated (apoC-III1) proteoforms. Higher apoC-III2 and apoC-III2/apoC-III1 ratios were associated with lower triglycerides and total cholesterol, and with lower small dense LDL. ApoC-III2/apoC-III1 was also higher in patients treated with PPAR-γ agonists [27].

Neu5Ac levels were markedly elevated in the breast milk of mothers whose infants had a low risk of obesity [29]. Neu5Ac from breast milk altered infant gut microbiota and bile acid metabolism, resulting in a distinct fecal bile acid profile in the high-Neu5Ac group, which was characterized by reduced levels of primary bile acids and elevated levels of secondary bile acids. Taurodeoxycholic acid 3-sulfate and taurochenodeoxycholic acid 3-sulfate were correlated with high breast milk Neu5Ac and low obesity risk in infants, and their associations with healthy growth were reproduced in mice colonized with infant-derived microbiota. Parabacteroides might be linked to bile acid metabolism and mediate between Neu5Ac and infant growth [29].

Overall, these findings highlight the potential of sialic acid as a biomarker for obesity. Studies have also reported that sialylation of several metabolically beneficial glycoproteins is reduced in obesity, which seems inconsistent with the elevated total sialic acid levels observed in serum. This discrepancy may be explained by inflammation-induced shedding or secretion of sialic acids as well as by increased production of sialic acid–rich, inflammation-responsive proteins, which could mask the decreased sialylation of specific glycoproteins. Therefore, future studies on the role of sialylation in metabolism should preferably focus on specific glycoconjugates rather than crude analysis of total sialic acid levels in serum.

## 3. Sialic Acids as Biomarkers for Diabetes and Insulin Resistance

Diabetes mellitus is a chronic metabolic disorder of hyperglycemia caused by either an absolute deficiency of insulin secretion (Type 1 diabetes, T1DM) or an impaired insulin sensitivity (Type 2 diabetes, T2DM). Millions of individuals around the world are suffering from diabetes and consequently contributing to significant mortality because of its complications, which include cardiovascular disease, diabetic retinopathy, and kidney disease. T1DM typically happens in younger patients and involves autoimmune destruction of insulin-secreting pancreatic beta cells, leading to absolute insulin deficiency. T2DM, which is more prevalent, is strongly associated with obesity, insulin resistance, and relative insulin deficiency. Studies found a profound relationship between sialic acids and diabetes development.

### 3.1. Sialic Acids in Type 1 Diabetes

In T1DM, there have been reports showing that total sialic acid is not elevated compared to non-diabetic controls, but correlations with cardiovascular risk factors such as triglycerides and total cholesterol were seen [30,31,32]. A ten-year follow-up study suggests that elevated serum sialic acid could be an early predictor of nephropathy in T1DM [33]. The study found that elevated serum sialic acid levels were observed approximately three years before the onset of persistent microalbuminuria. By studying the relationship between plasma sialic acid levels and insulin deficiency in various diabetic animal models, it was found that an elevated plasma sialic acid level is associated with marked insulin deficiency, rather than hyperglycemia per se [34]. In obese and lean mice treated with streptozotocin (STZ), there was a marked increase in serum sialic acid following insulin depletion, while hyperglycemia alone did not cause a similar elevation [34]. Interestingly, fasting was shown to increase plasma sialic acid in both lean and obese mice, possibly due to reduced insulin levels during fasting, further confirming the correlation between insulin deficiency and sialic acid [34]. The fact that plasma total sialic acid levels in patients with type 1 diabetes are relatively unchanged is probably due to the use of insulin for treatment [34]. To further prove this theory, we should focus on the plasma TSA levels in patients with newly diagnosed type 1 diabetes who have not yet received insulin treatment in future research.

### 3.2. Sialic Acids in Type 2 Diabetes

Different studies have consistently reported elevated TSA levels in T2DM patients, especially those with complications [16,35]. In a study on Kuwaiti T2DM patients, TSA levels were found to be significantly higher in diabetic patients compared to non-diabetic controls, and elevated levels were correlated with cardiovascular risk factors apolipoprotein B, LDL cholesterol, and triglycerides [16]. Other studies highlighted that serum N-acetylneuraminic acid was elevated in T2DM patients with both diabetic retinopathy and diabetic kidney disease, linking sialic acids to the pathogenesis of diabetes complications [35,36].

Insulin resistance, a common hallmark of obesity and T2DM, is closely linked to alterations in the sialylation status of adipose tissue and other insulin-sensitive tissues. One of the most extensively studied sialic acid-modified molecules is ganglioside GM3. Gangliosides are a major subclass of glycosphingolipids enriched in mammalian cell membranes, particularly in the nervous system. They are characterized by one or more sialic acid residues attached to their oligosaccharide chains [37]. They are essential in intercellular signaling, neural development, and disease pathogenesis [37]. GM3 ganglioside plays an important role in the modulation of inflammation and insulin signaling pathways. In omental adipose tissue of obese, insulin-resistant women, increased GM3 levels and the corresponding sialyltransferase ST3GAL5 were associated with adipocyte hypertrophy, macrophage infiltration, and inflammation [38]. These changes are consistent with findings in mouse models, where GM3 induced by inflammatory cytokines such as TNF-α has been shown to impair insulin signaling [39]. Specific GM3 molecular species also act as endogenous TLR4 ligands. In metabolic syndrome, a shift in GM3 species, with increased pro-inflammatory variants, correlates with disease progression, highlighting GM3’s dual role in inflammation and insulin resistance [40]. GM3-degrading sialidases like NEU3 were also reported to be involved in modulating insulin sensitivity. In obese animals, impaired NEU3 activity correlates with decreased liver and skeletal muscle insulin sensitivity. The restoration of NEU3 levels improves insulin signaling, again highlighting the importance of sialic acid metabolism in glucose homeostasis [41].

Furthermore, in TNF-α-induced insulin resistance, adipocytes exhibit the decreased sialylation of membrane glycoproteins, which correlates with reduced GLUT4 translocation to the plasma membrane and impaired glucose uptake [42].

Collectively, current evidence supports a strong association between altered sialylation and impaired insulin signaling, suggesting that altering sialylation of specific molecules is a potential target for the treatment of type 2 diabetes.

## 4. Molecular Insights into Sialic Acids and Obesity/Diabetes

### 4.1. Changes in IgG Glycoforms in Immune Dysfunction and Multiple Disorders

Immunoglobulin G (IgG) sialylation is critical in modulating immune responses and maintaining homeostasis. Our current understanding is that sialylation of the IgG Fc-region primarily affects its interactions with Fcγ receptors and immune modulation, particularly through the addition of α2,6-linked sialic acids [43,44]. Although Fab-region sialylation has also been observed, there is no strong evidence that sialylation of the IgG Fab region alters antigen specificity [43]. During autoimmune diseases such as rheumatoid arthritis, systemic lupus erythematosus, and inflammatory bowel disease, IgG molecules tend to lose the sialic acid residues, a condition referred to as hyposialylation [43]. The absence of terminal sialic acids in the IgG Fc-region increases its pro-inflammatory potential by increasing its affinity with activating Fc receptors, and decreasing its affinity with inhibitory Fc receptors, thereby leading to amplified immune responses and tissue damage [43].

Metabolic disorders such as diabetes and obesity are characterized by low-grade chronic inflammation, in which hyposialylated IgG is also present and potentially used as a biomarker of metabolic status. Profiling IgG glycosylation in a large cohort showed low galactosylation and sialylation, along with increased fucosylation, to be associated with poor metabolic health and chronic inflammation [45]. In accordance with these findings, central adiposity as measured by the android/gynoid (A/G) ratio and waist-to-height ratio is clearly associated with a pro-inflammatory IgG glycan profile, including reduced sialylation [46]. Emerging evidence suggests that IgG hyposialylation is associated with atherosclerosis, a chronic inflammatory disease that leads to the formation of arterial plaque [47]. Hyposialylated IgG enhances the activation of inflammatory pathways in endothelial cells, leading to vascular inflammation and plaque instability, which are central to the pathophysiology of atherosclerosis [47]. Extensive research has demonstrated that metabolic interventions such as low-calorie diets, bariatric surgery, and physical exercise can significantly shift IgG glycosylation [48,49,50,51]. For example, weight loss achieved by bariatric surgery led to a shift toward a younger IgG glycan profile, characterized by increased digalactosylation and sialylation [49]. A comparison trial of various diets in 1,850 individuals showed that, after an 8-week low-calorie diet, participants experienced a statistically significant reduction in agalactosylated IgG glycans and an increase in anti-inflammatory sialylated IgG glycans [50]. Mechanistically, hyposialylated IgG binds more efficiently to Fcγ receptors on endothelial cells, contributing to insulin resistance [47]. That is because the interaction of FcγRIIB with hyposialylated IgG interfered with insulin transcytosis in endothelial cells [47]. In a high-fat diet (HFD) model, FcγRIIB-deficient mice, either systemically or conditionally in endothelial cells, were protected from insulin resistance despite becoming obese, since glucose uptake was unimpaired [47].

There is still controversy about the effect of exercise on IgG glycosylation. A moderate pro-inflammatory glycan shift was observed in the IgG of obese individuals after three months of physical exercise [48]. A decrease in digalactosylated, monosialylated, and disialylated structures, as well as an increase in agalactosylated, asialylated, and core-fucosylated structures was reported [48]. Despite minimal weight loss and unchanged dietary habits of the participants, there was a slight shift in body composition (fat-to-muscle ratio). Therefore, it was hypothesized that this pro-inflammatory IgG profile may be an early response to the release of sequestered pro-inflammatory cytokines as fat stores begin to mobilize during the initial stages of metabolic remodeling [48]. Another study showed that rigorous exercise regimes with energy deficits induce a pro-inflammatory shift in IgG glycosylation, characterized by decreased galactosylation and sialylation and increased bisecting GlcNAc [52]. In contrast, intense exercise in healthy individuals led to an anti-inflammatory shift, with increased sialylation and galactosylation of IgG glycans [51]. These discrepancies highlight the importance of baseline fitness, metabolic state, and energy availability in determining how exercise influences IgG glycosylation and inflammation.

### 4.2. Regulation of Sialylation in Adipogenesis

Sialylation is also engaged in adipogenesis [53]. ST6GAL1, an enzyme that adds α2-6-linked sialic acid to glycoproteins, is downregulated in the visceral adipose tissues of obese mice [53]. Downregulation of ST6GAL1 is associated with increased adipocyte differentiation and body weight gain [53]. Overexpression of ST6GAL1 in adipocytes inhibits adipogenesis, at least in part by increasing the phosphorylation of focal adhesion kinase, an essential mediator of integrin signaling. Moreover, epigenetic modifications, such as DNA methylation, regulate ST6GAL1 expression during obesity and represent a potential therapeutic target for modulating adipogenesis [53].

Another study on muscle-derived progenitor cells showed that a reduction in α-2,3- and α-2,6-linked sialic acids on N-glycans promotes early adipogenic differentiation and repels myogenesis [54]. This reduction in sialylation at the cell surfaces altered cell-cell interactions and favors adipogenic pathways over myogenic ones [54]. Moreover, Liu et al. identified significant decreases in α2,6-sialylation, α1,6-fucosylation, and α1,6-mannosylation in both embryonic stem cells (ESCs) and parthenogenetic ESCs during adipogenesis [55]. Together, these findings suggest that downregulated sialylation is a major mechanism in inducing adipogenic differentiation, potentially contributing to obesity by enhancing adipocyte development, and hence may be an excellent target for therapeutic intervention in metabolic diseases.

### 4.3. Neuraminidases and Metabolic Dysfunction

Sialidases, also known as neuraminidases, are a group of enzymes that release sialic acid residues from glycoconjugates, such as glycoproteins, glycolipids, and oligosaccharides [56]. These enzymes are present in most organisms, from viruses and bacteria to mammals. By modulating cell surface sialylation levels, sialidases are involved in critical cellular activities such as cell communication, receptor activation and immune responses. Dysregulation of sialidase activity has been found linked to diverse diseases, including metabolic disorders, cancer, neurodegeneration, and infections [56,57]. In humans, four major sialidase isoenzymes, namely NEU1, NEU2, NEU3, and NEU4, are localized to distinct cellular compartments and exhibit specific substrate preferences and physiological roles [56].

Among these, NEU1 is the most abundant sialidase, primarily localized to lysosomes and the plasma membrane. NEU1 requires association with protective protein/cathepsin A (PPCA) for lysosomal targeting, catalytic activation, and stabilization [58]. In the absence of PPCA, NEU1 forms inactive oligomers and is functionally impaired. This PPCA–NEU1 interaction is essential for NEU1’s enzymatic role in the lysosome and is disrupted in lysosomal storage disorders such as galactosialidosis [58]. NEU1’s involvement in several metabolic diseases has been reviewed previously, highlighting its complex regulatory effects on insulin signaling, obesity, and non-alcoholic fatty liver disease (NAFLD) [59]. A study examining different mouse models of obesity demonstrated that acidic sialidase activity, corresponding to lysosomal NEU1, is aberrantly regulated across multiple tissues in obese mice [60]. In particular, NEU1 activity was observed to be elevated in epididymal white adipose tissue of obese mice and decreased in liver, whereas NEU1 mRNA was comparable in epididymal and kidney leaf fat but reduced in the liver of obese mice [60]. NEU1 also influences hepatic lipid metabolism, promoting lipid storage and contributing to the pathogenesis of NAFLD. Knockdown of NEU1 via miR-205 has been shown to ameliorate hepatic lipid accumulation in both in vitro and in vivo models [61]. However, further studies are needed to clarify the full impact of NEU1 on NAFLD.

**Figure 2:**
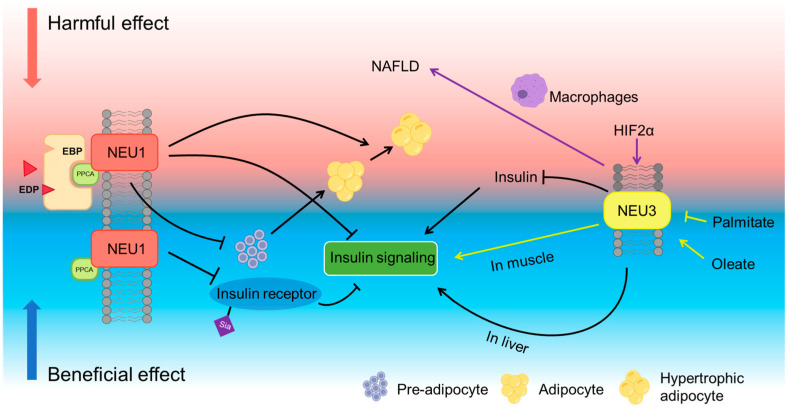
Controversial Roles of Neuraminidases in Metabolic Diseases. In the figure, the arrows indicate activation or upregulation, and the flat-ended lines indicate inhibition.

Studies by Pshezhetsky and Hinek have substantially advanced our understanding of the role of NEU1 in insulin signaling and metabolic regulation. Their work demonstrated that NEU1 desialylates insulin receptors (IRs) and insulin-like growth factor 1 receptors (IGF-1Rs), thereby enhancing IR activation and cellular responses (Figure 2) [62,63,64]. Evidence from Neu1-deficient mice shows impaired insulin sensitivity and glucose intolerance without significant changes in insulin production, suggesting that NEU1 primarily affects insulin action rather than secretion [62]. Insulin-induced AKT phosphorylation in cells from sialidosis patients with Neu1 gene deficiency was impaired [62]. Pharmacological induction of NEU1 activity, for example, by Ambroxol, was shown to restore insulin sensitivity in obese mice [63].

In addition to acting independently, NEU1 can also function as a part of the elastin receptor complex (ERC). Differently, when acting in persistent exposure to elastin-derived peptides (EDPs), NEU1 associated with ERC appears to interact with IR and inhibit its activation [65]. These findings suggest that NEU1 can act as a context-dependent modulator of insulin signaling. This study also found that chronic exposure to EDPs is associated with increased lipid accumulation in liver and adipose tissues [65]. However, activation of the NEU1-containing ERC by the stimulation of EDP can reduce lipogenesis in adipocytes, whereas ERC inhibitors may promote adipogenesis and restore adipocyte differentiation [59], indicating that the effects differ on precursor cells and mature adipocytes (Figure 2).

NEU3, another membrane-associated sialidase, predominantly acts on glycolipids. NEU3 has been reviewed for its involvement in obesity and diabetes, though its impact on insulin sensitivity and metabolic regulation remains controversial and context-dependent [66].

Pioneering work from Miyagi et al. demonstrates that NEU3 exerts tissue- and context-dependent control of insulin signaling [67,68,69]. NEU3 deficiency in mice increases insulin secretion under feeding conditions without altering blood glucose, and pharmacologic inhibition of sialidases enhances insulin release in isolated islets, indicating a regulatory role in β-cell activity [69]. In whole-body NEU3-overexpressing transgenic mice, insulin signaling is attenuated, with reduced phosphorylation of the insulin receptor and downstream targets such as IRS-1 and Akt, leading to glucose intolerance and fasting hyperglycemia. These mice also show insulin resistance and fasting hyperglycemia, phenocopying key features of type 2 diabetes [67].

Interestingly, the inhibitory effect of NEU3 on insulin signaling appears to be tissue-specific. Adenovirus-mediated hepatic overexpression of NEU3 surprisingly led to improved insulin sensitivity and enhanced glucose tolerance [68]. NEU3 remodeled hepatic gangliosides, increased basal and insulin-stimulated IRS-1 tyrosine phosphorylation [68].

Furthermore, NEU3 expression dynamically responds to nutritional signals, especially fatty acids. For instance, Lipina et al. demonstrated that saturated fatty acid palmitate suppresses NEU3 expression in skeletal muscle cells, impairing insulin signaling and while oleate preserves NEU3 levels and maintains insulin sensitivity, suggesting NEU3 acts as a lipid-responsive regulator of insulin action [41]. This dual effect underscores the complexity of NEU3’s action in metabolic homeostasis.

Moreover, a study using CCl_4_-induced liver damage showed that NEU3 inhibition reduced macrophage infiltration, pro-inflammatory cytokines and suppressed fibrosis markers [70], implicating it in the transition from steatosis to nonalcoholic steatohepatitis (NASH). Recent studies have also linked NEU3 to the gut-liver axis regulated by hypoxia-inducible factor-2α (HIF-2α). During obesity, HIF-2α activation in the intestine upregulates NEU3, enhancing ceramide levels through the salvage pathway and leading to hepatic lipid accumulation and liver inflammation. This suggests that NEU3 may contribute to hepatic steatosis not only through direct hepatic action but also by modulating nutrient uptake and lipid trafficking from the intestine to the liver [71].

In addition, NEU3 also regulates gastrointestinal barrier homeostasis and inflammation. In an acquired colitis model, TLR4-dependent induction of epithelial NEU3 desialylates nascent Mucin-2, rendering the colonic mucosal barrier susceptible to cathepsin-G proteolysis. Conversely, genetic or pharmacologic depletion of NEU3 preserves Mucin-2 and mitigates disease severity [72]. Complementary evidence further shows that Neu3 is responsible for desialylation and deficiency of intestinal alkaline phosphatase (IAP) during recurrent infection, whereas Neu3-null mice retain IAP and are protected from colitis [73], positioning NEU3 as a regulator of gut barrier integrity that is probably mechanistically linked to metabolic disease–associated GI inflammation.

NEU2 is a mammalian sialidase localized in the cytosol. Unlike NEU1 and NEU3, its expression in human tissues is generally low, but it has been linked to myogenic differentiation and cancer cell apoptosis. A Neu2 knockout mouse model showed that loss of NEU2 abrogates lipid metabolism, resulting in hypertriglyceridemia, fatty liver, impaired muscle differentiation and motor function, ultimately leading to obesity-like phenotypes [74]. NEU4 is a mammalian sialidase with broad glycoprotein/ganglioside specificity that localizes mainly to lysosomes and mitochondria. NEU4 contributes to neuronal function and lysosomal/autophagy pathways. It is downregulated in colorectal cancer and supports glioblastoma stem-cell survival, and it is upregulated in kidney fibrosis [75]. However, direct evidence linking NEU4 to metabolic disease is limited.

Taken together, these studies highlight the role of sialidases as key regulators of metabolic homeostasis. As such, targeting neuraminidases, particularly NEU1, NEU2, and NEU3, may offer novel therapeutic strategies for obesity, type 2 diabetes, and NAFLD. However, the role of sialidases in insulin signaling and lipid accumulation is sometimes controversial, particularly for NEU1 and NEU2 (Figure 2). Analysis of these studies reveals that sialidases have a wide range of target organs, but their effects likely vary across these organs. The current contrasting and complicated outcomes underscore the necessity of tissue- and molecule-specific neuraminidase targeting strategies to further illustrate their roles or to be used for therapeutic targets.

### 4.4. Sialyltransferase and Metabolic Dysfunction

Sialyltransferases are a family of glycosyltransferases responsible for catalyzing the transfer of sialic acid residues to glycoconjugates. They act primarily in the Golgi and generate the diverse sialylation patterns that are ultimately displayed on the cell surface, secreted proteins, or extracellular matrix. In humans, at least twenty distinct sialyltransferases have been identified and categorized into four prominent families based on their substrate preferences and linkage specificities, including ST3GAL1-6, ST6GAL1-2, ST6GALNAC1-6, and ST8SIA1-6 [76]. These enzymes contribute to a broad range of physiological processes, including cell-cell communication, immune response, cancer progression, and pathogen recognition.

**Figure 3:**
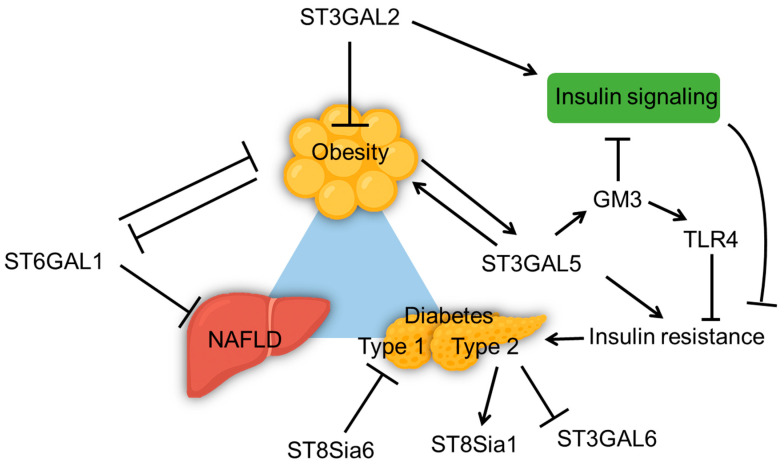
Altered Sialyltransferases in Obesity and Diabetes: Implicated in Disease Development. Sialyltransferases install terminal sialic acids in the Golgi that tune receptor function and immune tone. Dysregulation of specific sialyltransferases leads to organ-specific consequences in metabolism. Arrows indicate activation or upregulation, and flat-ended lines indicate inhibition.

ST6GAL1 is the key enzyme for adding α2,6-linked sialic acids to the galactose on IgG Fc and other glycoproteins. It is mainly synthesized in the hepatocytes and secreted into the circulation as a soluble enzyme [77]. Studies have found that ST6GAL1 expression is downregulated in the visceral adipose tissue of obese mice fed with HFD (Figure 3), and ST6GAL1 knockout results in significantly increased body weight and visceral fat of HFD mice [53]. Hepatic conditional knockout of St6gal1 displayed a reduction in α2,6-sialylation of liver and plasma glycoproteins in mice, which caused a compensatory increase in α2,3-sialylation, altered fucosylation, and branching of N-glycans in plasma proteins. As the hepatic ST6GAL1^−/−^ mice aged, they spontaneously developed NAFLD characterized by fat accumulation and inflammation in the liver, and a shift in macrophage populations from anti-inflammatory Kupffer cells to pro-inflammatory M1 macrophages. Interestingly, hepatic ST6GAL1 deficiency in mice did not exacerbate HFD-induced obesity [78]. These findings suggest that the loss of ST6Gal1 in hepatocytes disrupts liver homeostasis, triggers metaflammation, and promotes metabolic dysfunction [78], positioning ST6GAL1 as a protective metabolic modulator.

ST3GAL2 transfers α2,3-linked sialic acids to galactose preferentially on gangliosides GM1 and GD1b to form GD1a and GT1b. Mice lacking ST3GAL2, characterized by altered ganglioside composition in adipose tissue, developed late-onset obesity and insulin resistance on a standard diet. The ST3GAL2 deficiency disrupted insulin signaling by impairing insulin receptor-mediated phosphorylation in adipose tissue but not liver or skeletal muscle, leading to hyperglycemia and insulin resistance [79].

ST3GAL5 (GM3 synthase) catalyzes the synthesis of GM3 ganglioside, which is key to regulating insulin sensitivity and inflammation in metabolic diseases. Studies have shown that increased gene expression of ST3GAL5 and GM3 levels in adipose tissue and serum correlate with insulin resistance, especially in obese and diabetic conditions [38]. Elevated GM3 impaired insulin signaling by disrupting IR localization and function in lipid rafts [38]. GM3 also influences innate immune responses via Toll-like receptor 4 (TLR4) modulation. In obesity, specific GM3 species are up-regulated and enhance TLR4 signaling, thus impacting chronic inflammation associated with obesity and metabolic syndrome. This mechanism contributes to a pro-inflammatory environment, exacerbating insulin resistance and metabolic disorders [40,80]. ST3GAL5-deficient model mice exhibited improved insulin sensitivity and reduced high-fat diet-induced obesity [81,82]. ST3GAL5-knockout in mice and treatments that reduce GM3 levels in diabetic wound models enhance keratinocyte migration and proliferation by activating IR and IGF-1 receptors [83]. siRNA-based therapies targeting ST3GAL5 in diabetic wounds have also accelerated wound closure through improved cellular signaling [84]. Although both ST3GAL2 and ST3GAL5 belong to the α2,3-sialyltransferase family and participate in ganglioside biosynthesis, they exert opposing effects on insulin signaling and metabolic homeostasis. This contrast highlights how the balance of ganglioside species defines the metabolic outcome. As ganglioside biosynthesis proceeds from GM3 toward more complex forms such as GD1a and GT1b, the effects on insulin receptor signaling turn from inhibitory to facilitative. Inflammation could change the cell surface glycosylation pattern by modifying sialyltransferase expression as well. The reduction in α2,3-sialylation on adipocyte membrane proteins, associated with lower ST3GAL6 expression, occurred after TNF-α treatment to induce insulin resistance [46].

In pancreatic β cells, ST8SIA family enzymes shape immune tolerance and insulin secretion. Notably, β cell–specific overexpression of ST8Sia6 in NOD mice confers protection against autoimmune diabetes, as the generated α-2,8-disialic acid structures mitigate immune-mediated destruction and preserve glucose homeostasis [85]. Consistently, ST8Sia6 was also shown to attenuate hyperglycemia in STZ-induced diabetes models, underscoring its potential as a therapeutic target for type 1 diabetes [86]. In parallel, human islet studies identified ST8Sia1 as a marker of distinct β-cell subtypes with differential insulin secretory capacity and stress responses, implicating this enzyme in β-cell heterogeneity [87]. In type 2 diabetes, the proportion of ST8Sia1^+^ β cells often increases, and these cells exhibit higher basal insulin secretion and weakened glucose responses [87]. Together, these advances highlight that the ST8Sia family adds an additional layer of complexity to the role of sialyltransferases in obesity and diabetes, linking glycan remodeling with both immune modulation and intrinsic β-cell function.

In summary, dysregulation of specific SiaTs leads to organ-specific consequences, including metaflammation in liver, insulin resistance in adipose tissue, and β-cell dysfunction in pancreas (Figure 3). Moreover, different sialic acid linkages appear to exert distinct yet interconnected functions. Further investigation into the dynamic balance among α2-3, α2-6, and α2-8 sialylation is needed. Compensatory remodeling between linkage types has been observed, but its significance in metabolic disorders remains unclear. Integrating advanced glycoproteomics, single-cell analysis, and metabolic phenotyping techniques will help elucidate how this sialylation balance shapes systemic metabolic homeostasis.

### 4.5. Role of Siglecs in Metabolic Disorders

Siglecs (Sialic acid-binding ImmunoGlobulin-type LECtins) are a family of cell-surface receptors that play essential roles in immune regulation through their binding to sialic acid-containing glycans. These receptors are expressed predominantly on immune cells, such as macrophages, neutrophils, monocytes, B cells, dendritic cells, and subsets of natural killer and T cells, and serve as crucial checkpoints by distinguishing self from non-self. Structurally, Siglecs are defined by their extracellular sialic acid-binding immunoglobulin-like domains and distinct intracellular signaling motifs. Most Siglecs contain immunoreceptor tyrosine-based inhibitory motifs (ITIMs) that recruit phosphatases to inhibit immune activation, whereas activating Siglecs (e.g., human Siglec-14, -15, -16, and murine Siglec-H) associate with DAP12 to signal through immunoreceptor tyrosine-based activation motifs (ITAMs). This dual capacity places Siglecs as central players in maintaining immune homeostasis, and their dysregulation is associated with various pathological conditions [8]. Changes in siglecs expression or function are increasingly linked to metabolic disease risks and complications.

Siglec-3(or known as CD33), expressed primarily on myeloid cells, functions as an immune checkpoint by restraining pro-inflammatory cytokines like TNF-α. Under diabetic hyperglycemia, Siglec-3 expression of peripheral monocytes is reduced, leading to increased TNF-α production and enhanced pro-inflammatory activities [88]. In a diet-induced obesity model, CD33^+^ myeloid-derived suppressor cells (MDSCs) are increased within the tumor microenvironment, which contribute to tumor-promoting immunosuppression. Depletion of MDSCs significantly attenuates obesity-accelerated oral carcinogenesis [89].

Siglec-15, an activating Siglec, also links obesity to cancer progression. In a model of diet-induced obesity, transcription factor EB (TFEB) directly bound the Siglec-15 promoter in non-small-cell lung cancer (NSCLC) cells, driving its upregulation alongside glycolytic genes. This suppressed CD8^+^ T-cell expansion and function while sustaining regulatory T cells in the tumor microenvironment, accelerating NSCLC cell growth in obesity. Blocking TFEB reduced Siglec-15 expression, restored antitumor immunity, and enhanced the efficacy of PD-1 blockade in obese mice [90].

Siglec-7 is commonly known as an inhibitory receptor on NK cells, helping to regulate NK cell activity. In obese individuals, Siglec-7 levels on NK cells in obese individuals appear to be somewhat contradictory. It was reported that Siglec-7 expression increases in the overall NK cell population in obesity, but is reduced in the CD56^bright^ NK subset [91,92]. In the pancreas, Siglec-7 is expressed by β-cells, where it is markedly downregulated in both type 1 and type 2 diabetes. Siglec-7 restoration in diabetic islets reduces inflammatory cytokine production and preserves β-cell function, underscoring its direct protective role in diabetes [93].

In the aortic endothelium, hyperglycemia induces Siglec-9 ligand expression, driving co-cultured macrophages toward increased apoptosis and impaired phagocytosis [94]. The increase in Siglec-9 ligands in hyperglycemia may serve as a compensatory mechanism against vascular inflammation in diabetic angiopathy [94]. In vivo, overproduction of the ligands of Siglec-E (the murine functional ortholog of human Siglec-9), α2,8-disialic acids, alleviates hyperglycemia in the low-dose STZ-induced diabetes [86]. Conversely, genetic Siglec-E deficiency exacerbated adipose-tissue inflammation and worsened body weight and glycemia under a high-fat diet [95]. Sialic acid-dependent recognition of CD24 by Siglec-E recruits SHP-1, a phosphatase that inhibits inflammatory signaling. The CD24-Siglec-E pathway acts as a significant immune brake on diet-induced obesity, dyslipidemia, insulin resistance, and NASH [96].

Siglec-1 (sialoadhesin) has recently been implicated in type 1 diabetes pathogenesis [97]. A novel subset of Siglec-1^+^ monocytes with a strong interferon signature was identified in patients with pediatric type 1 diabetes and latent autoimmune diabetes in adults. These cells are enriched for chemokine receptors and T-cell chemoattractants, infiltrate pancreatic islets in NOD mice, and accelerate disease onset when adoptively transferred. Their frequency correlates positively with disease activity and may serve as a biomarker and therapeutic target for autoimmune diabetes [97].

Siglec-5 has been identified as a receptor on endothelial cells that mediates oxidized LDL transcytosis, promoting lipid accumulation in the vascular wall and foam cell formation [98]. This interaction accelerates atherosclerosis, particularly in diabetic patients, linking Siglec-5 to lipid-related cardiovascular risks in metabolic disease. Elevated plasma levels of Siglec-5 in diabetic patients are associated with critical limb ischemia, a severe form of peripheral arterial disease [99], suggesting its potential both as a pathogenic mediator and as a biomarker for severe vascular complications.

**Figure 4:**
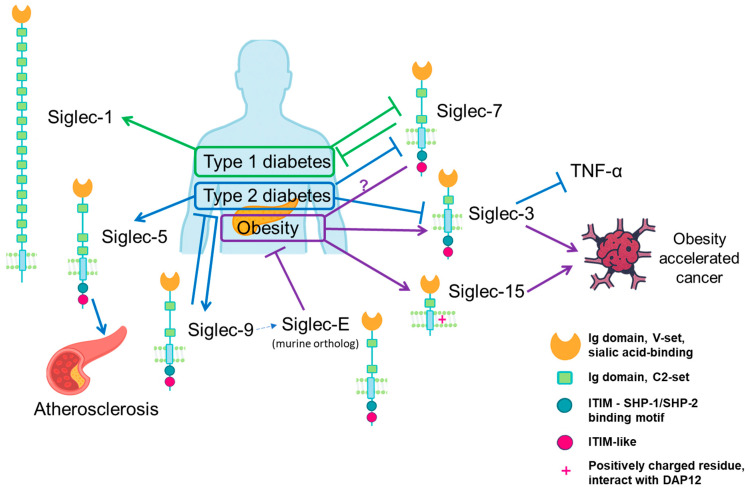
Siglec dysregulation across obesity and diabetes: altered sialic acid-Siglec axis shaping immunometabolic pathology. Arrows indicate activation or upregulation, and flat-ended lines indicate inhibition. Question marks represent un-certain regulatory relationships.

Together, current evidence positions Siglecs as modulators of both metabolic inflammation and its downstream complications (Figure 4). In obesity and diabetes, Siglec dysregulation occurs across multiple compartments including immune cells, vascular endothelium, adipose tissue, and even tumor cells. This contributes not only to impaired glucose and lipid metabolism but also to heightened risks of complications such as atherosclerosis and obesity-driven cancers. Restoring inhibitory signaling (e.g., Siglec-7, Siglec-E), suppressing pathogenic subsets (Siglec-1^+^ monocytes), or blocking tumor-promoting checkpoints (Siglec-15) could, in principle, modulate key immunometabolic pathways. However, most current data are correlative. Functional validation using tissue-specific knockout models is still limited. The molecular complexity of sialic acid biosynthesis and Siglec signaling across tissues highlights the need for deeper mechanistic research. Strengthening this foundation will be essential to determine whether Siglecs can ultimately be developed as viable therapeutic targets for obesity, diabetes, and their complications.

## 5. Therapeutic Interventions Targeting Sialic Acids

Several studies have investigated sialic acid–based therapies, including Neu5Ac supplementation, for obesity-related metabolic disorders. The use of sialic acid–rich substances such as Edible Bird’s Nest (EBN) as dietary interventions has also been explored for their metabolic benefits.

Neu5Ac supplementation showed benefits in HFD-fed rodent models, such as reducing serum lipid levels, attenuating insulin resistance, and preventing HFD-induced inflammation and oxidative stress [100,101]. A correlation between reduced IgG sialylation and higher systolic blood pressure was observed in obesity. ManNAc supplement, a precursor of sialic acid, has been shown to increase sialylation of IgG in HFD-induced obese mice [102]. This process prevented hypertension by normalizing IgG function and reducing endothelial FcγRIIB activation, thus supporting vascular health [102].

EBN is a traditional food rich in sialic acids, known for various health benefits like immune modulation, anti-oxidation, and metabolic support [103]. Multiple studies have reported EBN as a functional food with potential applications for treating metabolic disorders [104,105,106]. EBN supplementation in obese, HFD-fed mice reduced markers of inflammation and oxidative stress [105], displayed improved glucose and lipid metabolism [104], and attenuated hepatic lipid accumulation and steatosis [106], supporting healthier metabolic profiles.

Targeting sialidases to modulate sialic acid levels offers another therapeutic approach. Sialidase inhibitors such as DANA (2,3-dehydro-2-deoxy-N-acetylneuraminic acid) have been shown to reduce adipose tissue and liver inflammation in HFD mice [107]. Mice treated with DANA exhibited lower levels of inflammatory markers and improved glucose tolerance. In addition, they displayed less hepatic steatosis and reduced adipocyte hypertrophy [107].

## 6. Concluding Remarks

Dysregulated sialylation contributes to a range of metabolic impairments, including insulin resistance, chronic inflammation, and NAFLD. Defining the molecular mechanisms by which sialic acids regulate metabolic health and developing sialic acid–based therapies could open new treatment avenues. However, current studies remain limited, as most have focused on measuring total serum sialic acid levels. While it serves as an effective indicator of obesity-related inflammation, the method measures sialic acids from multiple bound forms across diverse molecules, including glycoproteins and glycolipids. Consequently, the results lack specificity, limiting their utility and the interpretation of underlying mechanisms. To precisely reveal the role of sialic acid in metabolic regulation and identify more specific biomarkers and therapeutic targets, future research must refine its approach, focusing on specific glycoconjugates, linkage, or derivatives.

Furthermore, key regulatory molecules in the sialic acid metabolic pathway, such as sialidases, sialyltransferases, and Siglecs, represent promising but underexplored therapeutic targets. However, existing research on these molecules is still insufficient, and the findings are occasionally contradictory. One potential reason for this is that current studies often focus on global analyses, overlooking the fact that these molecules often act on multiple target organs and may have distinct functions in different tissues. Therefore, future research should aim to provide deeper insights and focus on the development of targeted approaches directed at specific molecules and tissues. This strategy is expected to improve efficacy while minimizing adverse reactions.

Finally, rapid advances in glycosynthesis and glycomics/glycoproteomics are expected to transform our understanding of sialic acid biology. Integrating these tools will enable researchers to decode the “sialylation code” underlying metabolic adaptation and pave the way toward glycan-targeted strategies for metabolic disease prevention and therapy.

## Data Availability

No new data were created or analyzed in this study.

## Abbreviations

The following abbreviations are used in this manuscript:

BMI	Body mass index
CMAS	CMP-sialic acid synthetase
CRP	C-reactive protein
DANA	2,3-dehydro-2-deoxy-N-acetylneuraminic acid
EBN	Edible Bird’s Nest
EDP	Elastin-derived peptide
ERC	Elastin receptor complex
ESC	Embryonic stem cells
GM3	Monosialodihexosylganglioside (GM3 ganglioside)
HFD	High fat diet
HIF-2α	Hypoxia-inducible factor 2 alpha
IgG	Immunoglobulin G
IR	Insulin receptor
ITAMs	immunoreceptor tyrosine-based activation motifs
ITIMs	immunoreceptor tyrosine-based inhibitory motifs
LDL	Low density lipoprotein
MDSCs	Myeloid-derived suppressor cells
NAFLD	Non-alcoholic fatty liver disease
NASH	Non-alcoholic steatohepatitis
NEU	Neuraminidase
Neu5Ac	N-acetylneuraminic acid
SiaT	Sialyltransferase
Siglecs	Sialic acid-binding immunoglobulin-type lectins
STZ	Streptozotocin
T1DM	Type 1 diabetes
T2DM	Type 2 diabetes
TFEB	Transcription factor EB
TLR4	Toll-like receptor 4
TNF-α	Tumor necrosis factor alpha
TSA	Total sialic acids

## References

[1] Lewis A.L., Chen X., Schnaar R.L., Varki A., Varki A., Cummings R.D., Esko J.D., Stanley P., Hart G.W., Aebi M., Mohnen D., Kinoshita T., Packer N.H., Prestegard J.H. (2022). Sialic Acids and Other Nonulosonic Acids. Essentials of Glycobiology.

[2] Schauer R., Kamerling J.P. (2018). Exploration of the Sialic Acid World. Advances in Carbohydrate Chemistry and Biochemistry.

[3] Flynn R.A., Pedram K., Malaker S.A., Batista P.J., Smith B.A.H., Johnson A.G., George B.M., Majzoub K., Villalta P.W., Carette J.E. (2021). Small RNAs Are Modified with N-Glycans and Displayed on the Surface of Living Cells. Cell.

[4] Graziano V.R., Porat J., Ah Kioon M.D., Mejdrová I., Matz A.J., Lebedenko C.G., Chai P., Pluvinage J.V., Ricci-Azevedo R., Harrison A.G. (2025). RNA N-Glycosylation Enables Immune Evasion and Homeostatic Efferocytosis. Nature.

[5] Zhang N., Tang W., Torres L., Wang X., Ajaj Y., Zhu L., Luan Y., Zhou H., Wang Y., Zhang D. (2024). Cell Surface RNAs Control Neutrophil Recruitment. Cell.

[6] Chou H.-H., Hayakawa T., Diaz S., Krings M., Indriati E., Leakey M., Paabo S., Satta Y., Takahata N., Varki A. (2002). Inactivation of CMP-N-Acetylneuraminic Acid Hydroxylase Occurred Prior to Brain Expansion during Human Evolution. Proc. Natl. Acad. Sci. USA.

[7] Zhou J.Y., Oswald D.M., Oliva K.D., Kreisman L.S.C., Cobb B.A. (2018). The Glycoscience of Immunity. Trends Immunol..

[8] Duan S., Paulson J.C. (2020). Siglecs as Immune Cell Checkpoints in Disease. Annu. Rev. Immunol..

[9] Baumann A.-M.T., Bakkers M.J.G., Buettner F.F.R., Hartmann M., Grove M., Langereis M.A., de Groot R.J., Mühlenhoff M. (2015). 9-O-Acetylation of Sialic Acids Is Catalysed by CASD1 via a Covalent Acetyl-Enzyme Intermediate. Nat. Commun..

[10] Smith B.A.H., Bertozzi C.R. (2021). The Clinical Impact of Glycobiology: Targeting Selectins, Siglecs and Mammalian Glycans. Nat. Rev. Drug Discov..

[11] Wang Y., Jin D., Ren L., Wang N., Jia Y., Zheng Z., Cai W., Fu H., Li G. (2025). Sialylation Shields Glycoproteins from Oxidative Stress: Mechanistic Insights into Sialic Acid Oxidation and Structural Stability. J. Am. Chem. Soc..

[12] Zeng J., Liu Y., Dong C., Chong S., Liu Y., Bian Z., Chen X., Fan S. (2025). Sialyltransferase ST3GAL4 Directs a Dual Mechanism to Promote Pancreatic Ductal Adenocarcinoma Progression by Regulating Endoplasmic Reticulum Stress and Mitochondrial Homeostasis. Biochim. Biophys. Acta Mol. Basis Dis..

[13] Jones R.B., Dorsett K.A., Hjelmeland A.B., Bellis S.L. (2018). The ST6Gal-I Sialyltransferase Protects Tumor Cells against Hypoxia by Enhancing HIF-1α Signaling. J. Biol. Chem..

[14] Yerlikaya F.H., Toker A., Çiçekler H., Arıbaş A. (2015). The Association of Total Sialic Acid and Malondialdehyde Levels with Metabolic and Anthropometric Variables in Obesity. Biotech. Histochem..

[15] Yilmaz F.M., Yilmaz G., Savas Erdeve S., Dallar Y., Topkaya B.C., Yücel D. (2007). Serum Sialic Acid, Hs-CRP and Oxidative Stress Parameters in Obese Children. J. Pediatr. Endocrinol. Metab..

[16] Abdella N., Akanji A.O., Mojiminiyi O.A., Al Assoussi A., Moussa M. (2000). Relation of Serum Total Sialic Acid Concentrations with Diabetic Complications and Cardiovascular Risk Factors in Kuwaiti Type 2 Diabetic Patients. Diabetes Res. Clin. Pract..

[17] Akin L., Kurtoglu S., Muhtaroğlu S., Yikilmaz A., Kendirci M., Mazicioglu M. (2011). The Association of Serum Sialic Acid with Carotid Intima-Media Thickness and Anthropometric and Metabolic Parameters in Obese Children and Adolescents. Ann. Nutr. Metab..

[18] Rajappa M., Ikkruthi S., Nandeesha H., Satheesh S., Sundar I., Ananthanarayanan P.H., Harichandrakumar K.T. (2013). Relationship of Raised Serum Total and Protein Bound Sialic Acid Levels with Hyperinsulinemia and Indices of Insulin Sensitivity and Insulin Resistance in Non-Diabetic Normotensive Obese Subjects. Diabetes Metab. Syndr..

[19] Kurtul N., Akarsu E., Aktaran S. (2006). The Relationship between Serum Total Sialic Acid Levels and Adenosine Deaminase Activity in Obesity. Saudi Med. J..

[20] Crook M.A., Miell J., Ameerally P., Lumb P., Singh N., Russell-Jones D., Goldsmith L. (2003). Serum Sialic Acid, a Reputed Cardiovascular Risk Factor, Is Related to Serum Leptin Concentrations in Fijians. Clin. Chim. Acta.

[21] Browning L.M., Jebb S.A., Mishra G.D., Cooke J.H., O’Connell M.A., Crook M.A., Krebs J.D. (2004). Elevated Sialic Acid, but Not CRP, Predicts Features of the Metabolic Syndrome Independently of BMI in Women. Int. J. Obes. Relat. Metab. Disord..

[22] Crook M.A., Tutt P., Pickup J.C. (1993). Elevated Serum Sialic Acid Concentration in NIDDM and Its Relationship to Blood Pressure and Retinopathy. Diabetes Care.

[23] Hong Z., Zhou K., Wei Y., Ma B., Xie G., Zhang Z., Liang J. (2024). Associations of Plasma and Fecal Metabolites with Body Mass Index and Body Fat Distribution in Children. J. Clin. Endocrinol. Metab..

[24] Süer Gökmen S., Kazezoğlu C., Sunar B., Ozçelik F., Güngör O., Yorulmaz F., Gülen S. (2006). Relationship between Serum Sialic Acids, Sialic Acid-Rich Inflammation-Sensitive Proteins and Cell Damage in Patients with Acute Myocardial Infarction. Clin. Chem. Lab. Med..

[25] Suzzi S., Croese T., Ravid A., Gold O., Clark A.R., Medina S., Kitsberg D., Adam M., Vernon K.A., Kohnert E. (2023). N-Acetylneuraminic Acid Links Immune Exhaustion and Accelerated Memory Deficit in Diet-Induced Obese Alzheimer’s Disease Mouse Model. Nat. Commun..

[26] Harvie M.N., Pegington M., Mattson M.P., Frystyk J., Dillon B., Evans G., Cuzick J., Jebb S.A., Martin B., Cutler R.G. (2011). The Effects of Intermittent or Continuous Energy Restriction on Weight Loss and Metabolic Disease Risk Markers: A Randomized Trial in Young Overweight Women. Int. J. Obes..

[27] Koska J., Yassine H., Trenchevska O., Sinari S., Schwenke D.C., Yen F.T., Billheimer D., Nelson R.W., Nedelkov D., Reaven P.D. (2016). Disialylated Apolipoprotein C-III Proteoform Is Associated with Improved Lipids in Prediabetes and Type 2 Diabetes. J. Lipid Res..

[28] Kim T., Xie Y., Li Q., Artegoitia V.M., Lebrilla C.B., Keim N.L., Adams S.H., Krishnan S. (2021). Diet Affects Glycosylation of Serum Proteins in Women at Risk for Cardiometabolic Disease. Eur. J. Nutr..

[29] Ouyang R., Zheng S., Wang X., Li Q., Ding J., Ma X., Zhuo Z., Li Z., Xin Q., Lu X. (2023). Crosstalk between Breast Milk N-Acetylneuraminic Acid and Infant Growth in a Gut Microbiota-Dependent Manner. Metabolites.

[30] Kurtoğlu S., Atabek M.E., Muhtaroglu S., Keskin M. (2006). The Association of Serum Total Sialic Acid/Total Protein Ratio with Diabetic Parameters in Young Type 1 Diabetic Patients. Acta Diabetol..

[31] Crook M., Cartwright K., Lumb P., Worsley A. (2000). Serum Sialic Acid in Young Type-1 Diabetic Patients. Diabetes Res. Clin. Pract..

[32] Moussa M.A.A., Alsaeid M., Refai T.M.K., Abdella N., Al-Sheikh N., Gomez J.E. (2004). Association of Serum Sialic Acid with Cardiovascular Metabolic Risk Factors in Kuwaiti Children and Adolescents with Type 1 Diabetes. Metabolism.

[33] Yokoyama H., Jensen J.S., Myrup B., Mathiesen E.R., Rønn B., Deckert T. (1996). Raised Serum Sialic Acid Concentration Precedes Onset of Microalbuminuria in IDDM. A 10-Year Follow-up Study. Diabetes Care.

[34] Pickup J.C., Day C., Bailey C.J., Samuel A., Chusney G.D., Garland H.O., Hamilton K., Balment R.J. (1995). Plasma Sialic Acid in Animal Models of Diabetes Mellitus: Evidence for Modulation of Sialic Acid Concentrations by Insulin Deficiency. Life Sci..

[35] Tomofuji Y., Suzuki K., Kishikawa T., Shojima N., Hosoe J., Inagaki K., Matsubayashi S., Ishihara H., Watada H., Ishigaki Y. (2023). Identification of Serum Metabolome Signatures Associated with Retinal and Renal Complications of Type 2 Diabetes. Commun. Med..

[36] Cheeseman J., Kuhnle G., Stafford G., Gardner R.A., Spencer D.I., Osborn H.M. (2021). Sialic Acid As A Potential Biomarker for Cardiovascular Disease, Diabetes and Cancer. Biomark. Med..

[37] Schnaar R.L., Sandhoff R., Tiemeyer M., Kinoshita T., Varki A., Cummings R.D., Esko J.D., Stanley P., Hart G.W., Aebi M., Mohnen D., Kinoshita T., Packer N.H., Prestegard J.H. (2022). Glycosphingolipids. Essentials of Glycobiology.

[38] Wentworth J.M., Naselli G., Ngui K., Smyth G.K., Liu R., O’Brien P.E., Bruce C., Weir J., Cinel M., Meikle P.J. (2016). GM3 Ganglioside and Phosphatidylethanolamine-Containing Lipids Are Adipose Tissue Markers of Insulin Resistance in Obese Women. Int. J. Obes..

[39] Tagami S., Inokuchi J., Kabayama K., Yoshimura H., Kitamura F., Uemura S., Ogawa C., Ishii A., Saito M., Ohtsuka Y. (2002). Ganglioside GM3 Participates in the Pathological Conditions of Insulin Resistance. J. Biol. Chem..

[40] Inokuchi J.-I., Kanoh H. (2022). Pathophysiological Significance of GM3 Ganglioside Molecular Species with a Particular Attention to the Metabolic Syndrome Focusing on Toll-Like Receptor 4 Binding. Front. Mol. Biosci..

[41] Lipina C., Nardi F., Grace H., Hundal H.S. (2015). NEU3 Sialidase as a Marker of Insulin Sensitivity: Regulation by Fatty Acids. Cell Signal.

[42] Parker B.L., Thaysen-Andersen M., Fazakerley D.J., Holliday M., Packer N.H., James D.E. (2016). Terminal Galactosylation and Sialylation Switching on Membrane Glycoproteins upon TNF-Alpha-Induced Insulin Resistance in Adipocytes. Mol. Cell Proteom..

[43] Vattepu R., Sneed S.L., Anthony R.M. (2022). Sialylation as an Important Regulator of Antibody Function. Front. Immunol..

[44] Thaçi K., Anthony R.M. (2025). The Importance of IgG N-Glycosylation in Health, Disease, and Neonatal Hemochromatosis. Glycosci. Ther..

[45] Plomp R., Ruhaak L.R., Uh H.-W., Reiding K.R., Selman M., Houwing-Duistermaat J.J., Slagboom P.E., Beekman M., Wuhrer M. (2017). Subclass-Specific IgG Glycosylation Is Associated with Markers of Inflammation and Metabolic Health. Sci. Rep..

[46] Russell A.C., Kepka A., Trbojević-Akmačić I., Ugrina I., Song M., Hui J., Hunter M., Laws S.M., Lauc G., Wang W. (2019). Increased Central Adiposity Is Associated with Pro-Inflammatory Immunoglobulin G N-Glycans. Immunobiology.

[47] Tanigaki K., Sacharidou A., Peng J., Chambliss K.L., Yuhanna I.S., Ghosh D., Ahmed M., Szalai A.J., Vongpatanasin W., Mattrey R.F. (2018). Hyposialylated IgG Activates Endothelial IgG Receptor FcγRIIB to Promote Obesity-Induced Insulin Resistance. J. Clin. Investig..

[48] Šimunić-Briški N., Zekić R., Dukarić V., Očić M., Frkatović-Hodžić A., Deriš H., Lauc G., Knjaz D. (2023). Physical Exercise Induces Significant Changes in Immunoglobulin G N-Glycan Composition in a Previously Inactive, Overweight Population. Biomolecules.

[49] Greto V.L., Cvetko A., Štambuk T., Dempster N.J., Kifer D., Deriš H., Cindrić A., Vučković F., Falchi M., Gillies R.S. (2021). Extensive Weight Loss Reduces Glycan Age by Altering IgG N-Glycosylation. Int. J. Obes..

[50] Deriš H., Tominac P., Vučković F., Briški N., Astrup A., Blaak E.E., Lauc G., Gudelj I. (2022). Effects of Low-Calorie and Different Weight-Maintenance Diets on IgG Glycome Composition. Front. Immunol..

[51] Tijardović M., Marijančević D., Bok D., Kifer D., Lauc G., Gornik O., Keser T. (2019). Intense Physical Exercise Induces an Anti-Inflammatory Change in IgG N-Glycosylation Profile. Front. Physiol..

[52] Sarin H.V., Gudelj I., Honkanen J., Ihalainen J.K., Vuorela A., Lee J.H., Jin Z., Terwilliger J.D., Isola V., Ahtiainen J.P. (2019). Molecular Pathways Mediating Immunosuppression in Response to Prolonged Intensive Physical Training, Low-Energy Availability, and Intensive Weight Loss. Front. Immunol..

[53] Kaburagi T., Kizuka Y., Kitazume S., Taniguchi N. (2017). The Inhibitory Role of A2,6-Sialylation in Adipogenesis. J. Biol. Chem..

[54] Grassot V., Bouchatal A., Da Silva A., Chantepie S., Papy-Garcia D., Maftah A., Gallet P.-F., Petit J.-M. (2017). Heparan Sulfates and the Decrease of N-Glycans Promote Early Adipogenic Differentiation Rather than Myogenesis of Murine Myogenic Progenitor Cells. Differentiation.

[55] Liu W., Yan X., Liu W., Wang Y., Rao Y., Yu H., Cui J., Xie X., Sun M., Yin L. (2018). Alterations of Protein Glycosylation in Embryonic Stem Cells during Adipogenesis. Int. J. Mol. Med..

[56] Lipničanová S., Chmelová D., Ondrejovič M., Frecer V., Miertuš S. (2020). Diversity of Sialidases Found in the Human Body—A Review. Int. J. Biol. Macromol..

[57] Bourguet E., Figurska S., Fraączek M.M. (2022). Human Neuraminidases: Structures and Stereoselective Inhibitors. J. Med. Chem..

[58] Bonten E.J., Campos Y., Zaitsev V., Nourse A., Waddell B., Lewis W., Taylor G., d’Azzo A. (2009). Heterodimerization of the Sialidase NEU1 with the Chaperone Protective Protein/Cathepsin A Prevents Its Premature Oligomerization. J. Biol. Chem..

[59] Toussaint K., Appert-Collin A., Morjani H., Albrecht C., Sartelet H., Romier-Crouzet B., Maurice P., Duca L., Blaise S., Bennasroune A. (2022). Neuraminidase-1: A Sialidase Involved in the Development of Cancers and Metabolic Diseases. Cancers.

[60] Natori Y., Ohkura N., Nasui M., Atsumi G., Kihara-Negishi F. (2013). Acidic Sialidase Activity Is Aberrant in Obese and Diabetic Mice. Biol. Pharm. Bull..

[61] Hu Y., Ye H., Shi L.-X. (2019). MicroRNA-205 Ameliorates Lipid Accumulation in Non-Alcoholic Fatty Liver Disease through Targeting NEU1. Eur. Rev. Med. Pharmacol. Sci..

[62] Dridi L., Seyrantepe V., Fougerat A., Pan X., Bonneil E., Thibault P., Moreau A., Mitchell G.A., Heveker N., Cairo C.W. (2013). Positive Regulation of Insulin Signaling by Neuraminidase 1. Diabetes.

[63] Fougerat A., Pan X., Smutova V., Heveker N., Cairo C.W., Issad T., Larrivée B., Medin J.A., Pshezhetsky A.V. (2018). Neuraminidase 1 Activates Insulin Receptor and Reverses Insulin Resistance in Obese Mice. Mol. Metab..

[64] Arabkhari M., Bunda S., Wang Y., Wang A., Pshezhetsky A.V., Hinek A. (2010). Desialylation of Insulin Receptors and IGF-1 Receptors by Neuraminidase-1 Controls the Net Proliferative Response of L6 Myoblasts to Insulin. Glycobiology.

[65] Blaise S., Romier B., Kawecki C., Ghirardi M., Rabenoelina F., Baud S., Duca L., Maurice P., Heinz A., Schmelzer C.E.H. (2013). Elastin-Derived Peptides Are New Regulators of Insulin Resistance Development in Mice. Diabetes.

[66] Lipina C., Hundal H.S. (2015). Ganglioside GM3 as a Gatekeeper of Obesity-Associated Insulin Resistance: Evidence and Mechanisms. FEBS Lett..

[67] Sasaki A., Hata K., Suzuki S., Sawada M., Wada T., Yamaguchi K., Obinata M., Tateno H., Suzuki H., Miyagi T. (2003). Overexpression of Plasma Membrane-Associated Sialidase Attenuates Insulin Signaling in Transgenic Mice. J. Biol. Chem..

[68] Yoshizumi S., Suzuki S., Hirai M., Hinokio Y., Yamada T., Yamada T., Tsunoda U., Aburatani H., Yamaguchi K., Miyagi T. (2007). Increased Hepatic Expression of Ganglioside-Specific Sialidase, NEU3, Improves Insulin Sensitivity and Glucose Tolerance in Mice. Metabolism.

[69] Minami A., Fujita Y., Shimba S., Shiratori M., Kaneko Y.K., Sawatani T., Otsubo T., Ikeda K., Kanazawa H., Mikami Y. (2020). The Sialidase Inhibitor 2,3-Dehydro-2-Deoxy-N-Acetylneuraminic Acid Is a Glucose-Dependent Potentiator of Insulin Secretion. Sci. Rep..

[70] Pilling D., Martinez T.C., Gomer R.H. (2024). Inhibition of CCl4-Induced Liver Inflammation and Fibrosis by a NEU3 Inhibitor. PLoS ONE.

[71] Xie C., Yagai T., Luo Y., Liang X., Chen T., Wang Q., Sun D., Zhao J., Ramakrishnan S.K., Sun L. (2017). Activation of Intestinal Hypoxia-Inducible Factor 2α during Obesity Contributes to Hepatic Steatosis. Nat. Med..

[72] Yang W.H., Aziz P.V., Heithoff D.M., Kim Y., Ko J.Y., Cho J.W., Mahan M.J., Sperandio M., Marth J.D. (2023). Innate Mechanism of Mucosal Barrier Erosion in the Pathogenesis of Acquired Colitis. iScience.

[73] Yang W.H., Westman J.S., Heithoff D.M., Sperandio M., Cho J.W., Mahan M.J., Marth J.D. (2021). Neu3 Neuraminidase Induction Triggers Intestinal Inflammation and Colitis in a Model of Recurrent Human Food-Poisoning. Proc. Natl. Acad. Sci. USA.

[74] Oh M., Ha D.-I., Son C., Kang J.G., Hwang H., Moon S.B., Kim M., Nam J., Kim J.S., Song S.Y. (2022). Defect in Cytosolic Neu2 Sialidase Abrogates Lipid Metabolism and Impairs Muscle Function in Vivo. Sci. Rep..

[75] Glanz V.Y., Myasoedova V.A., Grechko A.V., Orekhov A.N. (2019). Sialidase Activity in Human Pathologies. Eur. J. Pharmacol..

[76] Li Y., Chen X. (2012). Sialic Acid Metabolism and Sialyltransferases: Natural Functions and Applications. Appl. Microbiol. Biotechnol..

[77] Jones M.B., Oswald D.M., Joshi S., Whiteheart S.W., Orlando R., Cobb B.A. (2016). B-Cell-Independent Sialylation of IgG. Proc. Natl. Acad. Sci. USA.

[78] Oswald D.M., Jones M.B., Cobb B.A. (2020). Modulation of Hepatocyte Sialylation Drives Spontaneous Fatty Liver Disease and Inflammation. Glycobiology.

[79] Lopez P.H., Aja S., Aoki K., Seldin M.M., Lei X., Ronnett G.V., Wong G.W., Schnaar R.L. (2017). Mice Lacking Sialyltransferase ST3Gal-II Develop Late-Onset Obesity and Insulin Resistance. Glycobiology.

[80] Inokuchi J., Kanoh H., Inamori K., Nagafuku M., Nitta T., Fukase K. (2022). Homeostatic and Pathogenic Roles of the GM3 Ganglioside. FEBS J..

[81] Inamori K., Ito H., Tamura Y., Nitta T., Yang X., Nihei W., Shishido F., Imazu S., Tsukita S., Yamada T. (2018). Deficient Ganglioside Synthesis Restores Responsiveness to Leptin and Melanocortin Signaling in Obese KKAy Mice. J. Lipid Res..

[82] Strekalova T., Veniaminova E., Svirin E., Kopeikina E., Veremeyko T., Yung A.W.Y., Proshin A., Tan S.Z.K., Khairuddin S., Lim L.W. (2021). Sex-Specific ADHD-like Behaviour, Altered Metabolic Functions, and Altered EEG Activity in Sialyltransferase ST3GAL5-Deficient Mice. Biomolecules.

[83] Wang X.-Q., Lee S., Wilson H., Seeger M., Iordanov H., Gatla N., Whittington A., Bach D., Lu J., Paller A.S. (2014). Ganglioside GM3 Depletion Reverses Impaired Wound Healing in Diabetic Mice by Activating IGF-1 and Insulin Receptors. J. Investig. Dermatol..

[84] Randeria P.S., Seeger M.A., Wang X.-Q., Wilson H., Shipp D., Mirkin C.A., Paller A.S. (2015). siRNA-Based Spherical Nucleic Acids Reverse Impaired Wound Healing in Diabetic Mice by Ganglioside GM3 Synthase Knockdown. Proc. Natl. Acad. Sci. USA.

[85] Choe J., Belmonte P., Crotts S., Nguyen T., Friedman D., Zastrow A., Rajcula M., Hammer B., Wilhelm C., Shapiro M.J. (2025). ST8Sia6 Overexpression Protects Pancreatic β Cells from Spontaneous Autoimmune Diabetes in Nonobese Diabetic Mice. J. Clin. Investig..

[86] Belmonte P.J., Shapiro M.J., Rajcula M.J., McCue S.A., Shapiro V.S. (2020). Cutting Edge: ST8Sia6-Generated α-2,8-Disialic Acids Mitigate Hyperglycemia in Multiple Low-Dose Streptozotocin-Induced Diabetes. J. Immunol..

[87] Dorrell C., Schug J., Canaday P.S., Russ H.A., Tarlow B.D., Grompe M.T., Horton T., Hebrok M., Streeter P.R., Kaestner K.H. (2016). Human Islets Contain Four Distinct Subtypes of β Cells. Nat. Commun..

[88] Gonzalez Y., Herrera M.T., Soldevila G., Garcia-Garcia L., Fabián G., Pérez-Armendariz E.M., Bobadilla K., Guzmán-Beltrán S., Sada E., Torres M. (2012). High Glucose Concentrations Induce TNF-α Production through the down-Regulation of CD33 in Primary Human Monocytes. BMC Immunol..

[89] Peng J., Hu Q., Chen X., Wang C., Zhang J., Ren X., Wang Y., Tao X., Li H., Song M. (2021). Diet-Induced Obesity Accelerates Oral Carcinogenesis by Recruitment and Functional Enhancement of Myeloid-Derived Suppressor Cells. Cell Death Dis..

[90] Zhang C., Zhou L., Li S., Zhao J., Meng X., Ma L., Wang Y., Li C., Zheng L., Ming L. (2022). Obesity Accelerates Immune Evasion of Non-Small Cell Lung Carcinoma via TFEB-Dependent Upregulation of Siglec-15 and Glycolytic Reprogramming. Cancer Lett..

[91] Naujoks W., Quandt D., Hauffe A., Kielstein H., Bähr I., Spielmann J. (2020). Characterization of Surface Receptor Expression and Cytotoxicity of Human NK Cells and NK Cell Subsets in Overweight and Obese Humans. Front. Immunol..

[92] Rosenstock P., Horstkorte R., Gnanapragassam V.S., Harth J., Kielstein H. (2017). Siglec-7 Expression Is Reduced on a Natural Killer (NK) Cell Subset of Obese Humans. Immunol. Res..

[93] Dharmadhikari G., Stolz K., Hauke M., Morgan N.G., Varki A., De Koning E., Kelm S., Maedler K. (2017). Siglec-7 Restores β-Cell Function and Survival and Reduces Inflammation in Pancreatic Islets from Patients with Diabetes. Sci. Rep..

[94] Zhang Y., Zheng Y., Li J., Nie L., Hu Y., Wang F., Liu H., Fernandes S.M., Zhong Q., Li X. (2019). Immunoregulatory Siglec Ligands Are Abundant in Human and Mouse Aorta and Are Up-Regulated by High Glucose. Life Sci..

[95] Rakib A., Mandal M., Al Mamun M.A., Kiran S., Yasmen N., Li L., Collier D.M., Jiang J., Park F., Singh U.P. (2025). Siglec-E Augments Adipose Tissue Inflammation by Modulating TRAF3 Signaling and Monocytic Myeloid-Derived Suppressor Cells during Obesity. Front. Immunol..

[96] Wang X., Liu M., Zhang J., Brown N.K., Zhang P., Zhang Y., Liu H., Du X., Wu W., Devenport M. (2022). CD24-Siglec Axis Is an Innate Immune Checkpoint against Metaflammation and Metabolic Disorder. Cell Metab..

[97] Guo M., Guo H., Zhu J., Wang F., Chen J., Wan C., Deng Y., Wang F., Xu L., Chen Y. (2024). A Novel Subpopulation of Monocytes with a Strong Interferon Signature Indicated by SIGLEC-1 Is Present in Patients with in Recent-Onset Type 1 Diabetes. Diabetologia.

[98] Jia X., Bai X., Yin Z., Zheng Q., Zhao Y., Lu Y., Shu Y., Wang Y., Zhang Y., Jin S. (2024). Siglec-5 as a Novel Receptor Mediates Endothelial Cells oxLDL Transcytosis to Promote Atherosclerosis. Transl. Res..

[99] Li J., Yang X., Wang X., Jia X., Wang Z., Deng A., Bai X., Zhu L., Li B., Feng Z. (2017). Siglec-5 Is a Novel Marker of Critical Limb Ischemia in Patients with Diabetes. Sci. Rep..

[100] Yida Z., Imam M.U., Ismail M., Ismail N., Ideris A., Abdullah M.A. (2015). High Fat Diet-Induced Inflammation and Oxidative Stress Are Attenuated by N-Acetylneuraminic Acid in Rats. J. Biomed. Sci..

[101] Kaburagi T., Otsuka Y., Oshiro S. (2023). Antiobesity Effect of N-Acetylneuraminic Acid by Enhancing Antioxidative Capacity in Mice Fed a High-Fat Diet. J. Med. Food.

[102] Peng J., Vongpatanasin W., Sacharidou A., Kifer D., Yuhanna I.S., Banerjee S., Tanigaki K., Polasek O., Chu H., Sundgren N.C. (2019). Supplementation with the Sialic Acid Precursor N-Acetyl-D-Mannosamine Breaks the Link Between Obesity and Hypertension. Circulation.

[103] Dai Y., Cao J., Wang Y., Chen Y., Jiang L. (2021). A Comprehensive Review of Edible Bird’s Nest. Food Res. Int..

[104] Zhang W., Zhu M., Liu X., Que M., Dekyi K., Zheng L., Zhang Y., Lv Y., Fan Q., Wang X. (2024). Edible Bird’s Nest Regulates Glucose and Lipid Metabolic Disorders via the Gut-Liver Axis in Obese Mice. Food Funct..

[105] Murugan D.D., Md Zain Z., Choy K.W., Zamakshshari N.H., Choong M.J., Lim Y.M., Mustafa M.R. (2020). Edible Bird’s Nest Protects Against Hyperglycemia-Induced Oxidative Stress and Endothelial Dysfunction. Front. Pharmacol..

[106] Yida Z., Imam M.U., Ismail M., Hou Z., Abdullah M.A., Ideris A., Ismail N. (2015). Edible Bird’s Nest Attenuates High Fat Diet-Induced Oxidative Stress and Inflammation via Regulation of Hepatic Antioxidant and Inflammatory Genes. BMC Complement. Altern. Med..

[107] Pilling D., Karhadkar T.R., Gomer R.H. (2021). High-Fat Diet-Induced Adipose Tissue and Liver Inflammation and Steatosis in Mice Are Reduced by Inhibiting Sialidases. Am. J. Pathol..

